# A qualitative study to understand the Duchenne muscular dystrophy experience from the parent/patient perspective

**DOI:** 10.1186/s41687-023-00669-6

**Published:** 2023-12-12

**Authors:** Victoria Brown, Elizabeth Merikle, Kelly Johnston, Katherine Gooch, Ivana Audhya, Linda Lowes

**Affiliations:** 1 Fortrea Inc. (formerly Labcorp Drug Development Inc.), 9711 Washingtonian Blvd., Suite 800, Gaithersburg, MD 20878 USA; 2 Sarepta Therapeutics, Inc., 215 First Street, Cambridge, MA 02142 USA; 3 Nationwide Children’s Hospital, 700 Children’s Dr, Columbus, OH USA

**Keywords:** Duchenne muscular dystrophy, Qualitative, Symptoms, Patient experience, Caregiver, Treatment expectations

## Abstract

**Background:**

Duchenne muscular dystrophy (DMD) is a rare, severe, fatal neuromuscular disease characterized by progressive atrophy and muscle weakness, resulting in loss of ambulation, decreased upper body function, and impaired cardiorespiratory function. This study aimed to generate qualitative evidence to describe the primary symptoms and impacts of DMD in ambulatory and non-ambulatory patients as reported by patient/caregiver dyads. Information was also gathered on expectations for future DMD treatments.

**Methods:**

Forty-six dyads (caregiver and patients with DMD aged 4 to 22 years) participated in 60-min semi-structured video interviews. Interview transcripts were analyzed using thematic analysis. Differences in experiences with DMD by ambulation status were examined.

**Results:**

Mean ages of ambulatory (n = 28) and non-ambulatory participants (n = 18) were 8.7 and 11.3 years, respectively, with an average age of diagnosis of 3.7 years (SD = 2.3). The primary symptoms reported by both groups were lack of strength (ambulatory: n = 28, 100.0%; non-ambulatory: n = 17, 94.4%) and fatigue (ambulatory: n = 24, 85.7%; non-ambulatory: n = 14, 77.8%). Physical function was the domain that was most impacted by DMD, with participants describing progressive decline of physical function due to loss of physical strength as the primary defining feature of the disease across all stages of ambulatory ability. For those who maintained ambulatory ability at the time of the interview, physical function impacts described impaired mobility (e.g., climbing stairs: n = 16, 57.1%; running: n = 13, 46.4%), impaired upper body function, in particular fine motor skills like holding a pen/pencil or buttoning clothes (n = 17, 60.7%), problem with transfers (e.g., getting off the floor: n = 10, 35.7%), and activities of daily living (ADLs; n = 15, 53.6%). For non-ambulatory participants, the functional impacts most frequently described were problems with transfers (e.g., getting in/out of bed: n = 13, 72.2%; getting in/out of chair or position in bed: both n = 10, 55.6%), impaired upper body function (reaching: n = 14, 77.8%), and ADLs (n = 15, 83.3%). Meaningful treatment goals differed by ambulatory status; for ambulatory participants, goals included maintaining current functioning (n = 20, 71.4%), improving muscle strength (n = 7, 25.9%), and reducing fatigue (n = 6, 22.2%). For non-ambulatory participants, these included increased upper body strength (n = 8, 42.1%) and greater independence in ADLs (n = 6, 31.6%). A preliminary conceptual model was developed to illustrate the primary symptoms and physical function impacts of DMD and capture their relationship to disease progression.

**Conclusion:**

This study contributes to the limited qualitative literature by characterizing impacts of physical limitations and symptoms of DMD on disease progression and thus providing insights into the lived experience with DMD. Differences in treatment goals were also identified based on ambulatory status. Taken together, these findings can help inform patient-centered measurement strategies for evaluating outcomes in DMD clinical research.

## Background

Duchenne muscular dystrophy (DMD) is a rare, severe, fatal neuromuscular disease affecting approximately 1 in 3500 to 5000 males born worldwide [1–3]. It is caused by mutations in the *DMD* gene on the X chromosome, leading to an absence of functional dystrophin protein. Disease progression is characterized by the continual atrophy and deterioration of skeletal and cardiac muscle [4]. While muscle damage is evident from birth, initial symptoms typically present around 3 to 5 years of age and include frequent falls and difficulties getting off the floor, walking, and climbing stairs [5–9]. Ambulation steadily decreases with most individuals becoming wheelchair dependent by early adolescence [10], followed by a decline in upper body functioning resulting in loss of independence. Serious comorbid complications, such as scoliosis and muscular contractures, often develop, and mortality is generally related to complications due to cardiomyopathy or respiratory compromise [11]. Life expectancy for individuals with DMD is typically limited to late 20 s or early 30 s [12].

No cure exists for DMD. The current standard of care for DMD focuses primarily on the management of symptoms and complications. However, survival of individuals with DMD has improved with the development of multidisciplinary care guidelines, improved management of cardiopulmonary dysfunction, and increased use of ventilatory support [13, 14]. Corticosteroids are the most common supportive treatment for DMD and are able to delay the loss of ambulation and may reduce the likelihood of needing spinal surgery to treat scoliosis [5, 14, 15]. More recently, exon-skipping treatments for patients with specific genotypes have been shown to attenuate pulmonary and ambulatory decline compared with mutation-matched natural history controls; however, only a small portion of the overall patient population (approximately 30%) can benefit from these advancements [16–22]. Emerging molecular and gene therapies designed to treat underlying disease process across the spectrum of possible genotypes in DMD are expected to change future DMD management [3]. In fact, the first gene therapy for the treatment of ambulatory individuals ages 4 through 5 years with DMD was recently approved by the United States Food and Drug Administration [23].

To date, only a handful of qualitative studies have described the symptoms and impacts of DMD on patients [22, 24–26]. These studies were limited to ambulatory boys and included relatively small sample sizes (< 10 patients and/or caregivers). The present study aimed to address these limitations and generate further qualitative evidence about the experience of living with DMD by including non-ambulatory participants as part of a larger sample. Caregivers and patients with DMD were interviewed together in dyads (pairs) to learn about symptoms, overall functioning, and the impacts of DMD on daily life. Additional information was gathered on what they desire from new treatments, including minimal benefits that would have a noticeable impact. Data from this study were used to develop a preliminary conceptual disease model for DMD that illustrates the predominant symptoms and impacts and their relationships to disease progression. This preliminary conceptual model can help to inform patient-centered clinical outcome assessment strategies for the evaluation of disease symptoms, impacts, and health-related quality of life in future DMD clinical trials.

## Methods

### Study design

This cross-sectional, qualitative interview study was conducted in the United States with 46 parent/patient dyads who met eligibility criteria (patients were males aged 4 years and older with a genetically confirmed DMD diagnosis, and caregivers were aged 18 years and older and a primary caregiver of a patient with a genetically confirmed DMD diagnosis). Participants were recruited via random stratified sampling from patients attending the muscular dystrophy and neuromuscular disorders clinics at Nationwide Children’s Hospital (NCH) or via email invitation through the Parent Project Muscular Dystrophy patient advocacy registry to enhance sample representativeness.

Recruitment was targeted to include participants along a full range of ambulation statuses based on the frequency of wheelchair use and walking ability. Patients were classified into three groups: ambulatory (walks all day and only uses a wheelchair for special outings), transitional (uses a wheelchair for at least part of the day for most days of the week but can still walk independently down a hall), and non-ambulatory (uses a wheelchair all the time and cannot walk unassisted). Ambulatory status was assessed by the Lowes Lab Ambulatory Status Algorithm during the screening process and confirmed during the interviews. Eligible participants who provided informed consent/assent participated in one 60-min semi-structured video interview and were remunerated $100 in the form of an electronic gift card upon completion of the interview. The study protocol and all participant-facing materials were approved by the NCH Institutional Review Board. Informed consent for participation and publication was obtained from study participants (or their parent or legal guardian for participants aged 4 to 17 years) before performing any study procedure. Assent was obtained from study participants aged 4 to 17 years. Participants were not directly informed about study findings but were indirectly informed by a presentation of the study results at the Muscular Dystrophy Association (MDA) 2022 scientific conference [27].

### Interview procedures

Trained qualitative researchers conducted interviews of the patient/caregiver dyad together using a semi-structured interview guide. The individual with DMD was asked to respond first, and the caregiver was asked to provide additional comments. If the individual with DMD did not provide detailed responses to questions, the interviewer probed for further details and directed questions to the caregiver to obtain more in-depth information. At the end of the interview, the caregiver was interviewed separately to allow for elaboration on responses without the patient present.

Open-ended questions were used to explore caregiver and patient perspectives of their experience with DMD and its impact on overall function and daily life. Attention was given to the terminology and language used to describe DMD symptoms and their associated impacts as well as the information regarding frequency, severity, variability, and burden of symptoms. Participants were also asked about their expectations of new treatments for DMD and how they would characterize treatment benefit.

### Analyses

Caregiver socioeconomic and demographic characteristics were summarized using descriptive statistics (mean, standard deviation, and frequency). All interviews were audio-recorded and professionally transcribed for qualitative analysis and de-identified prior to inclusion in the data set.

The transcripts were systematically analyzed via qualitative analysis software using thematic analysis methods adapted for patient-centered outcomes research [28] with features drawn from grounded theory, specifically the iterative process of constant comparison as new data were collected and analyzed [29–31]. An initial coding dictionary outlining each code and describing its meaning was developed based on the literature to guide the systematic analysis of the data. The first six transcripts were coded independently by two coders; discrepancies were reconciled prior to coding of additional transcripts. After reconciliation, the remaining transcripts were coded by one coder. The coding process was iterative, and the dictionary was updated throughout the analysis as new concepts and/or themes emerged. The research team evaluated the frequency of different symptoms and impacts discussed in the interviews and the relationships between them. Symptoms and impacts reported were then grouped into higher level concepts and themes and analyzed and interpreted based on ambulation status.

Data collection continued until saturation of concepts was identified. Concept saturation (i.e., the point at which no new relevant or important information regarding patient perception of a disease state is likely to be identified with continued data collection) is a widely accepted method for determining sample size in qualitative research on patient-reported outcomes [30, 32]. Saturation for the concept elicitation interviews was evaluated by developing a saturation grid with one row per participant to identify the point at which no new concepts were spontaneously elicited in the final interviews.

## Results

### Participant characteristics

Forty-two caregivers participated in the 46 dyad interviews, the majority of whom were mothers (87.8%) of the individuals with DMD; four caregivers had more than 1 child with DMD and participated in more than one dyad interview. The caregiver characteristics are shown in Table 1. Most caregivers were White (92.7%), had at least some college (95.1%), and were employed at the time of the interview (95.1%).

**Table 1:** Caregiver demographics (N = 42)*

Characteristic	%	Mean (SD)	Range
Age (years)		43.6 (8.7)	31–78
Relationship to child			
Mother	87.8		
Father	7.3		
Grandmother	2.4		
Grandfather	2.4		
Ethnic origin			
White	92.7		
Hispanic/Latino	2.4		
Native American/American Indian	2.4		
Asian/Pacific Islander	2.4		
Education level			
Grade 12 or less	0.0		
High school/GED	4.9		
Some college	43.9		
College graduate	19.5		
Graduate degree	31.7		
Marital status			
Married	82.9		
Divorced	9.8		
Single	4.9		
Widowed	2.4		
Currently employed			
Caregiver	95.1		
Spouse	80.5		
Has health insurance	63.4		
Number of children with DMD cared for		1.1 (0.38)	1–3

*Four caregivers had more than one child with DMD

More than half of the patient participants were classified as ambulatory (n = 28; 60.9%), of which 10 were initially classified as “transitional” based on the screening criteria. Analysis of the interview data found no systematic differences between the described symptom or impact experiences of the ambulatory and transitional participants, and thus findings for both groups are reported in aggregate. The average age of ambulatory participants was 8.7 years (standard deviation [SD] = 3.35, range 4–17) and average age of non-ambulatory participants was 11.3 years (SD = 3.27, range 10–22). The average age of DMD diagnosis for all patients was 3.7 years (SD = 2.3).

### Current symptoms and impacts of DMD

**Fig. 1:**
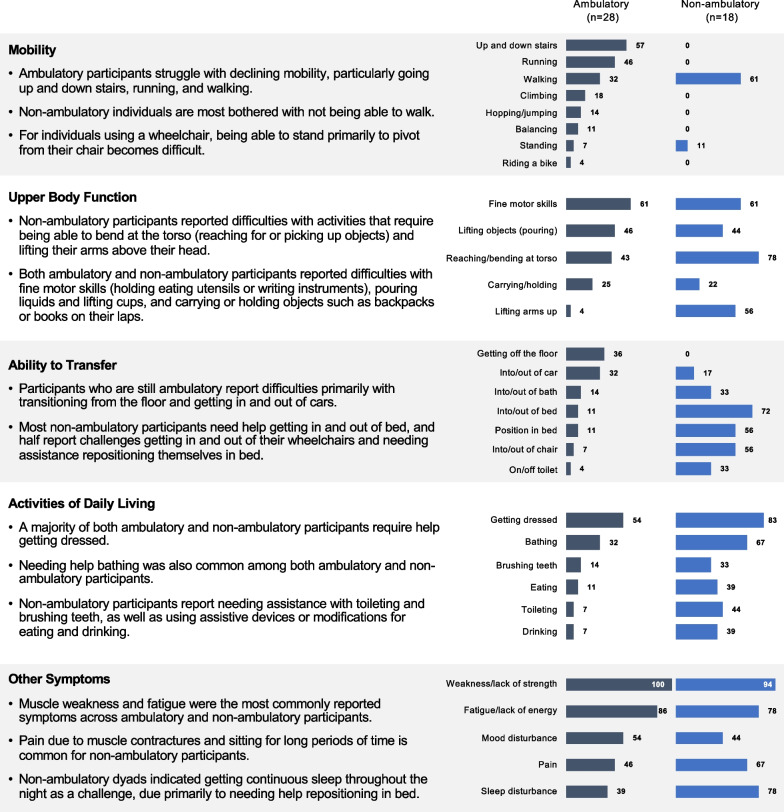
Frequency of symptoms and impacts reported by ambulatory status (N = 46)

Participants were asked to describe the current symptoms and impacts of DMD they experience in a typical day. These are presented in Fig. 1 and grouped into higher level concepts including *lower limb mobility* (incorporates problems with climbing stairs, running, and walking), *upper body function* (includes problems with fine motor skills, bending at torso, lifting objects, and others), *activities of daily living* (ADLs; e.g., bathing, getting dressed, toileting), *physical transfers* (pertaining to getting in and out of bed, getting in and out of a car, and sitting down), and *other disease symptoms* (referring to symptoms such as muscle weakness, fatigue, pain, and sleep disturbance). The qualitative analysis of current symptoms and impacts found the physical function domain was the primary domain of health-related quality of life impacted by DMD across all patients, as evidenced by the below results.

#### Ambulatory participants: most prevalent symptoms and impacts

Almost all ambulatory dyads reported at least one difficulty with mobility, including going up and down stairs (n = 16, 57%), running (n = 13, 46%), and walking (n = 9, 32%), which in turn impacted their ability to engage in activities they want to do, such as participation with their peers. One caregiver observed, “I would say his physical ability to be outdoors and play with other kids. He can’t really play with other kids as I know he would like.” For younger ambulatory individuals, the main concern was about the future impact of declining mobility as reflected by this caregiver: “I think just physical limitations and he’s 6, he’s in kindergarten, kids are still relatively kind but just looking into the future, I know that that is something that will continually probably impact him more as he gets older and he declines.”

Additionally, a large proportion also experienced difficulties with upper body function, including fine motor skills (n = 17, 61%). It is possible that problems with fine motor skills, like using a pencil, may be a result of a muscle weakness due to underlying DMD pathophysiologic processes affecting peripheral skeletal muscles not previously characterized or studied. Other challenges associated with upper body function included lifting/pouring (n = 13, 46%) and reaching for objects (n = 12, 43%), which limits the individual’s ability to contribute to household activities or the need for the caregiver to modify the task. One caregiver reported, “I need to look around to see like what he would pick-up. I mean I guess he likes to be helpful so he will try to grab like a grocery bag from the car and carry it in. He’ll take a couple steps and then just say no.”

Most reported difficulties with transfers, primarily getting off the floor (n = 10, 36%) and in and out of cars (n = 9, 32%) due to poor core strength, as well as requiring assistance with ADLs, such as getting dressed (n = 15, 54%) and bathing (n = 9, 32%).

All ambulatory dyads (n = 28, 100%) reported difficulties with weakness or lack of strength and most reported tiring easily or lacking energy (n = 24, 86%). Moreover, the tiredness seemed to result from the individual’s inability to sustain short-term physical activities, such as playing with siblings or peers. One caregiver observed, “[Name] would be able to play for 15 min and then you’d see him sitting on the side, just resting because he just got too tired.” Finally, for the majority of ambulatory individuals (n = 15, 54%), the array of physical difficulties they experienced had a negative impact on their mood. One caregiver reported, “Frustration levels I think recently. Sometimes he almost seems to get down on himself, depressed because of things.”

#### Non-ambulatory participants: most prevalent symptoms and impacts

For non-ambulatory participants, the symptoms and impacts reported were associated with impaired upper body function, including difficulties with reaching for objects (n = 14, 78%), fine motor skills such as holding a pencil or turning pages in a book (n = 11, 61%), lifting arms above the head (n = 10, 56%), and lifting objects (n = 8, 44%), which resulted in patients needing additional assistance from caregivers or adaptive devices. One caregiver indicated, “I would say he probably could lift a little cup with stuff in it with his Rexarm on. He uses a Rexarm…It’s an arm that assists when lifting a fork or spoon.”

Similarly, most non-ambulatory participants reported needing assistance with transfers, such as getting in and out of bed (n = 13, 72%), getting in and out of chairs (n = 10, 56%), and being positioned when in bed (n = 10, 56%), which further limited their independence. To overcome these challenges with transfers, some reported using assistive devices (e.g., Hoyer lift) or employing personal support workers. One non-ambulatory individual described his process for getting in and out of the shower: “With the sling, [Name] sits me in the shower chair, then pick my legs up and pull the sling out completely so it doesn’t get wet. Then I get the shower wheel in because it’s got wheels. I wheel into the shower, go to the ledge, get a shower and come back out, climb in, put the sling back under which takes four or five minutes because it’s a little hard but it’s not too hard.”

These limitations in upper body function resulted in all non-ambulatory participants needing assistance with at least one ADL, including getting dressed (n = 15, 83%), bathing (n = 12, 67%), toileting (n = 8, 44%), and eating and drinking (n = 7, 39%). As one non-ambulatory individual reported, “Showering, using the bathroom and just doing things with my arms, trying to get them above my head and getting clothes on. I mostly need help for everything now.” Two-thirds (n = 12, 66%) reported that needing assistance bathing and/or toileting was a major burden of living with DMD.

The majority of non-ambulatory dyads also described sleep problems (n = 14, 78%), primarily as a result of needing to be repositioned in bed. As one caregiver reported, “He can’t roll over or turn to get himself comfortable, so he has trouble falling asleep, and then being in one position for too long, he’ll wake up and I’ll have to come turn him and then it takes him a bit to fall back asleep.” Similarly, most reported experiencing fatigue (n = 14, 78%), which was attributed to sleep problems and tiredness from physical exertion as described by one individual with DMD: “I might get tired if I’ve been doing a lot and then I just feel exhausted.” For almost half of the non-ambulatory individuals, the loss of independence due to their physical limitations had a negative impact on their mood. As one caregiver reported, “He just gets frustrated if there’s something he can’t do that he wants to do. Asking for help sometimes is frustrating for him.”

#### Most bothersome challenges of living with DMD

Regardless of ambulatory status, the most bothersome challenges of living with DMD were reported to be mobility restrictions as well as the emotional and social impacts to patients that resulted from their physical function limitations. Approximately half of the ambulatory dyads reported the inability to keep up physically with their peers (n = 15, 54%) and/or mobility restrictions (n = 14, 50%). These challenges often had a negative emotional impact on the individual living with DMD, as stated by one caregiver, “I mean not being able to play with his friends—keep up with his friends, and that does upset him sometimes.” Similarly, among the non-ambulatory dyads, lack of mobility (n = 10, 56%) and inability to keep up with peers (n = 5, 28%) were reported to be the biggest challenges, which often resulted in negative emotions and feeling different from peers and limited their ability to participate in social activities, play, and sports. One non-ambulatory individual indicated that he wanted to be “Able to walk or just a sense of normalcy. I want to be like my brothers, walking, running, playing sports.”

### Treatment goals

The majority of ambulatory dyads (n = 20, 71%) reported that they would be satisfied with a new treatment that maintained their current level of functioning or stabilized the disease progression (i.e., not progress to loss of ambulation). As one caregiver noted, “I’d be most looking for stabilization, keep him as close to as he is now. Improvement is fantastic, but in this disease, stabilization is—would be a huge win.” In contrast, the majority of non-ambulatory dyads (n = 13, 72%) wanted to see an improvement in their mobility despite acknowledging this desire might not be realistic. One DMD individual noted, “I know this is unrealistic, but just maybe just fixing my mobility, but I doubt that’ll ever happen.”

Almost a quarter of participants in both groups (ambulatory: n = 8, 29%; non-ambulatory: n = 4, 22%) spontaneously reported that their expectation of a new treatment for DMD was one that slowed disease progression. For some dyads, a treatment that slowed progression was not enough improvement, but as noted by one caregiver, this was at a minimum what would be required of a treatment: “At least slow down the progression. I mean it would be nice for him to be able to walk again but at least just to have him—a longer life.”

### Treatment benefits

Participants were asked what small changes or improvements from a new treatment would make a meaningful difference in their lives. For ambulatory dyads, it would be better mobility (e.g., not tripping and falling, less muscle tightness) (n = 9, 33%), improved strength and/or less muscle weakness (n = 7, 26%), and improved endurance and/or more energy (n = 6, 22%). One caregiver observed, “It would be huge just to not trip and fall as much. Being able to run a little better, go upstairs easier; that’s where sometimes it’s a little tough just to see the extra efforts that go into those simple things that the rest of us do without thinking.”

Non-ambulatory dyads reported a desire for improved strength and/or less muscle weakness (n = 8, 42%) and more independence in carrying out ADLs (n = 6, 32%). One caregiver reported, “Just getting his arms stronger enough to feed himself with no difficulty.” Improvements to mobility (e.g., being able to stand) were also desired treatment benefits (n = 5, 26%).

### Conceptual model of DMD

**Fig. 2:**
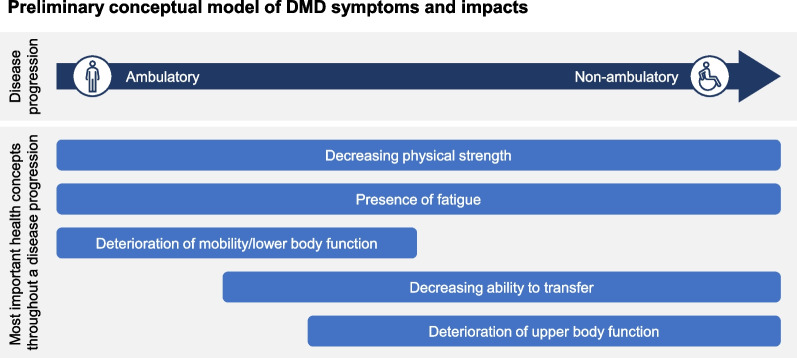
DMD conceptual model

Data from the qualitative interviews informed the development of a preliminary conceptual disease model for DMD (Fig. 2). The model includes ambulatory and non-ambulatory phases of DMD and displays the most important health concepts for patients as the disease progresses. Deteriorating physical strength was experienced throughout all phases of disease progression. Similarly, presence of fatigue was an important symptom early on in the disease progression during the ambulatory phase, and continued to be a problem in non-ambulatory patients. Difficulty with mobility was important in early stages of disease progression until loss of ambulation, at which time the ability to transfer became an increasing challenge to independence. Finally, decreasing upper body function begins to appear as individuals become less ambulatory and becomes the primary difficulty in the non-ambulatory phase.

## Discussion

This research contributes to the qualitative literature on the experience of people with DMD by summarizing findings from a large qualitative sample of both ambulatory and non-ambulatory individuals. The objectives were to obtain a richly textured understanding of the symptoms and impacts due to DMD experienced by patients. This study utilized a dyad approach, where a patient and their caregiver were interviewed together in order to ensure a rich understanding of the patient DMD experience. The dyad approach draws upon the individual patient and caregiver’s experiences with DMD as well as experiences that may be more likely to emerge together than in an individual context. By including non-ambulatory individuals, we gained a deeper understanding of the symptoms and impacts affecting DMD individuals in later stages of the disease and what they value most compared with individuals who are still ambulatory. Furthermore, regulatory guidance to approve new therapies, particularly in rare diseases that can present heterogeneously, relies on the patient’s perspective, which feeds into developing patient-reported outcome measures that can help determine what patients and their caregivers consider to be meaningful change.

While many of the concepts explored in this study are consistent with what has been reported previously in the literature [22, 24–26], this study identified additional novel insight into the impairment of upper extremity function in ambulatory as well as non-ambulatory individuals, supporting the notion of early and progressive involvement of upper extremity muscles in DMD alongside lower extremity muscles. Future research against non-DMD controls will need to add further evidence to corroborate this finding. Analyses to characterize the experience of individuals with DMD and investigate differences across ambulation statuses suggest very few differences in terms of overarching challenges and difficulties. In particular, when considering current symptom presentation, the most common challenges or difficulties across ambulation status were related to declining upper body function, lack of strength, and fatigue/tiredness. Further, keeping up with peers, endurance, and issues of mobility were reported by patients to be the most bothersome challenges associated with living with DMD, illustrating the psychosocial and emotional burdens associated with the disease.

The patient-centered conceptual model illustrates salient issues reported by participant dyads in this study. It corresponds with a framework previously developed by Williams et al. [26] for ambulatory individuals, which highlights the relationships between muscle weakness, reduced physical functioning, fatigue, and the resulting impacts on daily activities. Data from the current study offer broader insight into the mediating effect of ambulatory state on these relationships by including experiences of non-ambulatory individuals. For example, tiredness and fatigability experienced by ambulatory participants in our study were likely related to functional impairment of muscles, including progressive muscle weakness and breakdown, as opposed to a more systemic fatigue due to poor night’s sleep, reported as a challenge more frequently later in the disease.

When queried about what they would like to see in a new treatment, ambulatory dyads were more likely to be satisfied by maintaining current levels of functioning, particularly in mobility; these stabilizing benefits in lower extremity muscles would in turn allow for maintenance of muscle strength and less fatigue. Non-ambulatory dyads, on the other hand, desired increased strength primarily in their upper extremities, which would allow them to maintain or improve their independence when it comes to ADLs. These findings align with Peay et al. [31] wherein parents of younger patients (ambulatory) with DMD placed a higher value on skeletal muscle functioning to preserve mobility, encourage independence, and improved peer interactions. Parents of older DMD children described the muscle benefits as important in preserving self-care and improving quality of life.

Although this study provides new insights into the experience of DMD across ambulatory statuses, it also has limitations. Interviewing the caregiver in the presence of the child may have inadvertently influenced the child’s reporting. The study sample was predominantly white (n = 38, 92%), which limits generalizing to other races and socioeconomic levels, factors that are known to influence how individuals perceive health status and quality of life. Finally, participants at more advanced and severe stages of DMD were underrepresented. Given that the average age of non-ambulatory patients in this study was 11.3 years, one must take caution in extrapolating these findings to older non-ambulatory patients. Including more patients with advanced DMD should be a consideration for future qualitative studies to better understand the impact of the disease in late stages. Lastly, fatigue was not differentiated between muscular fatigue and systemic fatigue, and future studies should consider alternative approaches to evaluation of fatigue in DMD.

## Conclusions

This study contributes to the limited qualitative literature by characterizing impacts of physical limitations and symptoms of DMD throughout disease progression and thus providing insights into the lived experience with DMD. Differences in treatment goals were also identified based on ambulatory status. Further, the study results illustrated the tremendous psychological and social burdens of the disease, which are distal to declining physical function. The preliminary conceptual disease model illustrates most commonly reported health concepts and their evolution across the spectrum of ambulatory function, which provides a framework for patient-reported outcomes in future DMD studies. Taken together, these findings can help to inform patient-centered measurement strategies for evaluating outcomes in DMD clinical research.

## Data Availability

Qualified researchers may inquire by contacting the corresponding author.

## Abbreviations

ADL: Activities of daily living
DMD: Duchenne muscular dystrophy
MDA: Muscular Dystrophy Association
NCH: Nationwide Children’s Hospital
SD: Standard deviation

## References

[1] Emery AE (1991). Population frequencies of inherited neuromuscular diseases—a world survey. Neuromuscul Disord.

[2] Mendell JR, Rodino-Klapac LR, Sahenk Z (2013). Eteplirsen for the treatment of Duchenne muscular dystrophy. Ann Neurol.

[3] Sun C, Shen L, Zhang Z, Xie X (2020). Therapeutic strategies for Duchenne muscular dystrophy: an update. Genes (Basel).

[4] Falzarano MS, Scotton C, Passarelli C, Ferlini A (2015). Duchenne muscular dystrophy: from diagnosis to therapy. Molecules.

[5] Duan D, Goemans N, Takeda S, Mercuri E, Aartsma-Rus A (2021). Duchenne muscular dystrophy. Nat Rev Dis Primers.

[6] Jones HR, De Vivo DC, Darras BT (2003). Neuromuscular disorders of infancy, childhood, and adolescence: a clinician's approach.

[7] Mercuri E, Bonnemann CG, Muntoni F (2019). Muscular dystrophies. Lancet.

[8] Peverelli L, Testolin S, Villa L (2015). Histologic muscular history in steroid-treated and untreated patients with Duchenne dystrophy. Neurology.

[9] Timonen A, Lloyd-Puryear M, Hougaard DM (2019). Duchenne muscular dystrophy newborn screening: evaluation of a new GSP® neonatal creatine kinase-MM kit in a US and Danish population. Int J Neonatal Screen.

[10] Birnkrant DJ, Bushby K, Bann CM (2018). Diagnosis and management of Duchenne muscular dystrophy, part 2: respiratory, cardiac, bone health, and orthopaedic management. Lancet Neurol.

[11] Munot P (2022) BMJ best practice: muscular dystrophies. https://bestpractice.bmj.com/topics/en-us/969. Accessed 2 Jun 2023.

[12] Ryder S, Leadley RM, Armstrong N (2017). The burden, epidemiology, costs and treatment for Duchenne muscular dystrophy: an evidence review. Orphanet J Rare Dis.

[13] Birnkrant DJ, Bushby K, Bann CM (2018). Diagnosis and management of Duchenne muscular dystrophy, part 3: primary care, emergency management, psychosocial care, and transitions of care across the lifespan. Lancet Neurol.

[14] Lebel DE, Corston JA, McAdam LC, Biggar WD, Alman BA (2013). Glucocorticoid treatment for the prevention of scoliosis in children with Duchenne muscular dystrophy: long-term follow-up. J Bone Joint Surg Am.

[15] Khan N, Eliopoulos H, Han L (2019). Eteplirsen treatment attenuates respiratory decline in ambulatory and non-ambulatory patients with Duchenne muscular dystrophy. J Neuromuscul Dis.

[16] Iff J, Bungey G, Paine A, et al (2021) Delay of loss of ambulation with eteplirsen versus standard of care in Duchenne muscular dystrophy. In: Presented at the 2021 muscular dystrophy association virtual clinical and scientific conference, 15−18 Mar 2021

[17] Iff J, Gerrits C, Zhong Y (2022). Delays in pulmonary decline in eteplirsen-treated patients with Duchenne muscular dystrophy. Muscle Nerve.

[18] McDonald CM, Shieh PB, Abdel-Hamid HZ (2021). Open-label evaluation of eteplirsen in patients with Duchenne muscular dystrophy amenable to exon 51 skipping: PROMOVI trial. J Neuromuscul Dis.

[19] Mendell JR, Khan N, Sha N (2021). Comparison of long-term ambulatory function in patients with Duchenne muscular dystrophy treated with eteplirsen and matched natural history controls. J Neuromuscul Dis.

[20] Mitelman O, Abdel-Hamid HZ, Byrne BJ (2022). A combined prospective and retrospective comparison of long-term functional outcomes suggests delayed loss of ambulation and pulmonary decline with long-term eteplirsen treatment. J Neuromuscul Dis.

[21] Servais L, Mercuri E, Straub V (2022). Long-term safety and efficacy data of golodirsen in ambulatory patients with Duchenne muscular dystrophy amenable to exon 53 skipping: a first-in-human, multicenter, two-part, open-label, phase 1/2 trial. Nucleic Acid Ther.

[22] Staunton H, Trennery C, Arbuckle R (2021). Development of a clinical global impression of change (CGI-C) and a caregiver global impression of change (CaGI-C) measure for ambulant individuals with Duchenne muscular dystrophy. Health Qual Life Outcomes.

[23] ELEVIDYS (delandistrogene moxeparvovec-rokl) injection, for intravenous use [package insert]. Sarepta Therapeutics, Inc., Cambridge, MA (2023)

[24] Iff J, McKee S, Johnson C (2022). Conceptual models of the patient experience of Duchenne muscular dystrophy constructed from a qualitative interview study with caregivers. Value Health.

[25] Powell PA, Carlton J (2023). A comprehensive qualitative framework for health-related quality of life in Duchenne muscular dystrophy. Qual Life Res.

[26] Williams VN, McManus BM, Brooks-Russell A (2022). A qualitative study of effective collaboration among nurse home visitors, healthcare providers and community support services in the United States. Health Soc Care Community.

[27] Brown V, Merikle E, Johnston K, Audhya I, Gooch K, Lowes L (2022) A qualitative study to understand the Duchenne muscular dystrophy experience from the caregiver/patient perspective. In: Poster presentation at the Muscular Dystrophy Association (MDA) conference, Nashville, 13–16 Mar 2022

[28] Bryant A, Charmaz K, Cooper H (2012). Grounded theory and psychological research. Handbook of research methods in psychology.

[29] Strauss ACJ (1998). Basics of qualitative research: techniques and procedures for developing grounded theory.

[30] Cheng KKF, Clark AM (2017). Qualitative methods and patient-reported outcomes: measures development and adaptation. Int J Qual Methods.

[31] Landrum Peay H, Fischer R, Tzeng JP (2019). Gene therapy as a potential therapeutic option for Duchenne muscular dystrophy: a qualitative preference study of patients and parents. PLoS ONE.

[32] United States Food and Drug Administration (2022) Patient-focused drug development: methods to identify what is important to patients: guidance for industry, food and drug administration staff, and other stakeholders. https://www.fda.gov/regulatory-information/search-fda-guidance-documents/patient-focused-drug-development-methods-identify-what-important-patients. Accessed 15 Sept 2023

